# Exosomes from mmu_circ_0001052-modified adipose-derived stem cells promote angiogenesis of DFU via miR-106a-5p and FGF4/p38MAPK pathway

**DOI:** 10.1186/s13287-022-03015-7

**Published:** 2022-07-23

**Authors:** Zun-Hong Liang, Nan-Fang Pan, Shi-Shuai Lin, Zhi-Yang Qiu, Ping Liang, Jun Wang, Zhi Zhang, Yun-Chuan Pan

**Affiliations:** 1 Department of Burn and Skin Repair Surgery, Hainan General Hospital (Hainan Affiliated Hospital of Hainan Medical University), No.19, Xiuhua Road, Xiuying District, Haikou, 570311 Hainan Province People’s Republic of China; 2 Department of Burn Plastic Surgery, Guangzhou Red Cross Hospital Affiliated to Jinan University, No.396, Tongfu Middle Road, Guangzhou, 510632 Guangdong Province People’s Republic of China

**Keywords:** Diabetic foot ulcer, mmu_circ_0001052, miR-106a-5p, FGF4/p38MAPK pathway, Angiogenesis

## Abstract

**Background:**

Diabetic foot ulcer (DFU) is a chronic infectious disease caused by diabetes mellitus (DM). Angiogenesis plays the decisive role in wound healing of DFU. Adipose-derived stem cells (ADSCs) can ameliorate angiogenesis in DFU by exosomes. This study aims to determine the mechanism of exosomes from mmu_circ_0001052-modified ADSCs in angiogenesis of DFU.

**Methods:**

HUVECs were induced by high glucose and mice stimulated using STZ injection during high-fat feeding, which were treated with exosomes derived from mmu_circ_0001052-modified ADSCs. Real-time PCR determined the expression of gene and western blot determined protein levels. Proliferation, migration, apoptosis and angiogenesis of HUVECs were studied by MTT assay, transwell test, flow cytometry and tube formation experiment, respectively. Histological lesion of wound was determined by HE staining.

**Results:**

The expression of circ_0001052 was upregulated in ADSCs and miR-106a-5p elevated in high glucose-induced HUVECs. Exosomal mmu_circ_0001052 significantly accelerated wound healing in mice with DFU. Also, exosomal mmu_circ_0001052 evoked the reduction of miR-106a-5p and the elevation of FGF4 in high glucose-induced HUVECs and wound tissue of DFU mice. Exosomal mmu_circ_0001052 was determined to sponge miR-106a-5p that targeted FGF4 in DFU. In high glucose-induced HUVECs, exosomal mmu_circ_0001052 inhibited apoptosis and miR-106a-5p expression, and meanwhile promoted proliferation, migration, angiogenesis and expressions of FGF4, VEGF and p-p38/p38, which were reversed by miR-106a-5p elevation.

**Conclusion:**

Mmu_circ_0001052 in ADSCs-derived exosomes promote angiogenesis of DFU via miR-106a-5p and FGF4/p38MAPK pathway.

**Graphical Abstract:**

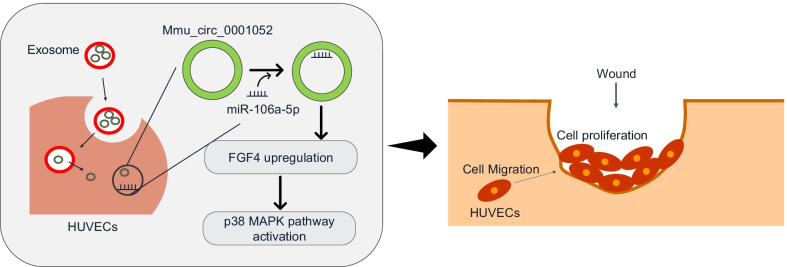

**Supplementary Information:**

The online version contains supplementary material available at 10.1186/s13287-022-03015-7.

## Background

Diabetic foot ulcer (DFU) is, the global health problem, a chronic infectious disease caused by diabetes mellitus (DM). According to statistic, the global incidence of DFU is 6.3% [1]. A prospective study [2] showed that DFU resulted in unsatisfactory clinical outcome and poor prognosis. On the one hand, DFU can cause foot deformity and increase skin pressure during walking [3]. On the other hand, it might raise the risk of limb invasive infection, which led to lower limb amputation [4]. Therefore, it is necessary to promote wound healing when patients with DM have DFU.

Angiogenesis plays the decisive role in wound healing of DFU. Angiogenesis is the process of existing blood vessels forming new blood vessels. It is not only a natural process of biological growth and development, but provides nutrients and oxygen for the injured site to promote wound healing [5]. During angiogenesis, endothelial cells stimulated by extracellular matrix proliferate rapidly and migrate to the stimulation site, which finally induces the formation of new blood vessels and vascular networks because of cells accumulation [6]. High glucose might directly inhibit the normal angiogenesis through endothelial dysfunction and the imbalance among angiogenesis related factors [7], which means that angiogenesis restoration is contributed to wound healing in DFU. Given in the key role of endothelial cells in angiogenesis, mountains of studies report that mesenchymal stem cells (MSCs) are instrumental in improving endothelial dysfunction. MSCs have the potential role in regulating proliferation and inflammatory [8, 9]. A study [10] suggested that MSCs-derived exosomes promote angiogenesis through regulating miR-30b in endothelial cells, which implies that MSCs could ameliorate angiogenesis in DFU by exosomes.

Circular RNA, a highly conservative non-coding RNA, is abundantly expressed in eukaryotic cells. It is involved in angiogenesis regulation via exosomes in diabetic wound healing [11]. Circular RNA HIPK3 (circRNA HIPK3) is a highly conserved non-coding RNA that mediated the vascular dysfunction and metabolic disorder induced by DM [12, 13]. CircRNA HIPK3 was determined to inhibit pyroptosis and cellular damage in ischemic disease via FOXO3a [14]. Mmu_circ_0001052 is a homolog of circular RNA HIPK3 showing the potential regulation in diabetic wound healing via its abnormal level in HUVECs stimulated by high glucose. CircRNA plays crucial role in cellular process that it competed with mRNA via sponging miRNA. MiR-106a-5p emerges as the critical role for tumor angiogenesis and endothelial dysfunction [15, 16]. Importantly, there was an elevated expression of miR-106a-5p in cell model of diabetes. Fibroblast growth factor 4 (FGF4), one member of fibrosis growth factor family, is involved in embryonic development, proliferation and differentiation [17–19]. A study [20] indicated that FGF4 promoted differentiation of MSCs into hepatocytes via p38 MAPK pathway.

MiR-106a-5p was predicted to bind to FGF4 mRNA or mmu_circ_0001052. There still was no research how the mmu_circ_0001052-miR-106a-5p-FGF4 mRNA-p38 MAPK axis and p38 MAPK pathway works on wound healing in DFU. Therefore, exosomes from autologous MSCs were applied for treating high glucose-induced HUVECs or mice with DFU in this study, which aimed to verify the mechanism of mmu_circ_0001052-miR-106a-5p-FGF4 mRNA network in angiogenesis of DFU.

## Methods

### ADSCs

ADSCs were isolated from adipose tissue in healthy BALB/c mice. The isolation of ADSCs referred to the study reported by Li et al. [21]. We characterized ADSCs via microcopy and flow cytometry (Beckman coulter, USA). The adipogenic differentiation of ADSCs was induced by the fixed Dulbecco’s Modified Eagle’s Medium (DMEM) with 10% fetal bovine serum (FBS, Gibco, USA), 10 μmol/L insulin (Solarbio, China), 200 μmol/L indomethacin (National institutes for food and drug control, China), 0.1 μmol/L dexamethasone (Solarbio, China) and 0.5 mmol/L isobutylmethylxanthine (Solarbio, China). ADSCs were cultured with DMEM containing 10% fetal bovine serum (FBS, Gibco, USA), 0.1 μmol/L dexamethasone (Solarbio, China), 50 μmol/L ascorbate-2-phosphate (Lookchem, China) and 10 mmol/L β-glycerophosphate (Seebio, China) to induce the osteogenic differentiation of these stem cells. After 14 days, ADSCs were stained with Oil-Red O (Solarbio, China) and alizarin red (Solarbio, China) to determine the differentiation. The overexpression vector of mmu_circ_0001052 (oe-mmu_circ_0001052) based on lentiviral was obtained from Sangon Biotech (Shanghai, China). 1 × 10^6^ of ADSCs were cultured in 6-well plates with FBS-free DMEM for 24 h and transfected by 100 μL of over-mmu_circ_0001052 mixture (with MOI of 50 and 1 × 10^8^ TU/mL) via CTS LV-MAX Transfection kit (ThermoFisher, MA, USA). The vector contained the gene coding green fluorescent protein, so the transfection efficiency could be observed by calculating fluorescence intensity via fluorescence microscopy. The exosomes were collected after 48-h transfection.

### Isolation and characterization of exosomes

We isolated the exosomes derived from ADSCs according to a previous study [11]. Briefly speaking, ADSCs in good condition were cultured with FBS-free endothelial cell growth medium (EGM)-2MV (Lonza, Switzerland). After 2 days, the exosomes in the medium were extracted by differential ultracentrifugation. After that, the protein content of PBS-resuspended exosomes was measured by BCA protein kit (Abcam, Cambridge, UK) and the exosomes were stored at − 80℃ for the next experiments. The characterization of exosomes was determined by western blot and transmission electron microscopy. ADSCs-derived exosomes contain various of protein and RNA that have biological activity.

### HUVECs

HUVECs were purchased from ATCC. HUVECs were cultured with DMEM containing 10% FBS which was replaced by DMEM containing 1% FBS before high glucose induction. HUVECs were stimulated with DMEM containing 33 mmol/L glucose for 48 h. For the control of high glucose stimulation, HUVECs were incubated in DMEM supplemented with 5 mmol/L glucose for 48 h. To investigate the role of ADSCs-derived exosomal mmu_circ_0001052 in high glucose-induced HUVECs, HUVECs were co-incubated with exosomes (20 μg/mL) derived from over-mmu_circ_0001052-modified ADSCs for 24 h. We used PKH26 (MINI26, Sigma Aldrich, USA)-labeled exosomes for co-incubation to distinguish external exosomes from secreted by cells themselves. Moreover, HUVECs were transfected by miR-106a-5p (3.33 μg) mimics and/or oe-circ_0001052 (100 μL) via lipofectamine 2000 (Invitrogen, USA). HUVECs were incubated in 6-well plates supplemented with FBS-free DMEM for 24 h before transfection. After 48-h-transfection, we determined the transfection efficiency using real-time PCR.

### Mice with DFU

We established the mice model of DFU according to the previous study of Li [22]. In general, male BALB/c mice (Shanghai Laboratory Animal Center of Chinese Academy of Sciences, China) with 5 weeks age and 19.39 ± 1.56 g weight were treated with 0.45% streptozotocin (Sigma, USA, 45 mg/kg) and feed with high-fat diet. We choosed these mice with the blood glucose level at 16.7 mmol/L to establish DFU model. There were 62 mice with DM. Then, we used the sterile punch to make wounds. Finally, exosomes derived from mmu_circ_0001052-modified ADSCs (exosomes + mmu_circ_0001052 group) or exosomes from vector-modified ADSCs (exosome + vector group) were subcutaneously injected. 25 μL of exosomes (200 μg in 100 μL PBS) were subcutaneously injected at 4 sites around the wound. The control group was not treated with exosomes. Wound tissues were collected at 3, 7 and 14 days after wounding. This study, following the Nation Institutes of Health Guide for the Laboratory Animals Care and Use, was carried out with the approval of hospital ethics committee, and Ethical approval No.:Med-Eth-Re [2021] 192.

### Dual-luciferase reporter gene

Firstly, the 3’-UTR of FGF4 and mmu_circ_0001052, containing the predicted binding site of miR-106a-5p, was amplified via PCR. Then, the recombinant luciferase reporter plasmids contained the potential miR-106a-5p binding site sequences of the FGF4 and mmu_circ_0001052. Finally, these plasmids were transfected into cells, respectively, and the luciferase intensity was detected using a double luciferase reporter gene detection system (Promega).

### RNA Binding protein immunoprecipitation

Starbase3.0 was used to predict the sequence information of mmu_circ_0001052 and miR-106a-5p, miR-106a-5p and FGF4. Magna RIPTM RNA-binding protein co-immunoprecipitation kit (Millipore) was used for co-immunoprecipitation. Cell lysate was treated with RIP buffer containing magnetic beads conjugated with human anti-Ago2 antibody (Millipore, Billerica, MA, USA), or negative control IgG. Beads were washed with wash buffer, and the complexes were incubated with 0.1% SDS/proteinase K to remove proteins. qRT-PCR assay was carried out afterward.

### Real-time PCR

The total RNA was extract by Trizol reagent (Invitrogen, USA). The OD value at 260 nm and 280 nm was determined. All-in-One™ miRNA qRT-PCR Detection System (GeneCopoeia, China) was used for PCR. The primers of mmu_circ_0001052, miR-106a-5p and FGF4 were designed and synthesized by GeneCopoeia. The reaction system and program were referenced from user manual. The internal reference genes were U6 and GAPDH. The relative expression was calculated by following equation: the relative expression = 2^−(ΔCt text gene−ΔCt internal reference gene)^, in which the Ct was cycle threshold. Table 1 lists these primers. And the full sequence of mmu_circ_0001052 was in the Additional file 1: Annex 1.

**Table 1:** Primers

Gene	Forward primer (5′ → 3′)	Reverse primer (5′ → 3′)
Mmu_circ_0001052	GGA TCG GCC AGT CAT GTA TC	ACC GCT TGG CTC TAC TTT GA
miR-106a-5p	TGC AGT AGA TCT CAA AAA GCT ACC	CCT TGG CCA TGT AAA AGT GC
FGF4	CCA TAG AGC TTG CCC TTG CT	GAA CCC TGG CCC TTT ATC CC
GAPDH	CGG AAT TCG TGA AGC TCG GAG TCA ACG G	CGG GAT CCC AGG AGC GCA GGG TTA GTC A
U6	ATTGGAACGATACAGAGAAGATT	GGAACGCTTCACGAATTTG

### Western blot

Firstly, ground samples were extracted by RIPA lysis buffer (Solarbio, China) and the concentration of protein in extract was measured by BCA protein kit (Abcam, Cambridge, UK). Then, the protein was transferred to PVDF membrane (Invitrogen, USA) after SDS-PAGE electrophoresis. The membrane was incubated with antibodies including FGF4 (ab106355, 1 μg/mL), VEGF (ab150375, 1:10000), p38 (ab170099, 1:1000) and p-p38 (ab178867, 1:1000) o before the secondary antibody (1:10000). Finally, Pierce™ ECL Western Blotting Substrate (Thermo Scientific, Beijing, China) was applied for blots visualization. All antibodies were purchased from Abcam.

### MTT assay

Before MTT assay, HUVECs were cultured into 96-well plate. The cell viability (or proliferation) was measured via MTT Assay Kit (Abcam, Cambridge, UK) after seeded 0, 12, 24, 48 and 72 h, respectively. Enzyme reader evaluated the OD value at 570 nm of the solution.

### Wound healing assay

HUVECs were seeded into 12-well culture plates supplemented with DMEM with 10% FBS at 37 °C until the confluence of HUVECs increased to 80%. Next, HUVECs were scratched with a 10-μL pipette tip after different treatments and subsequently incubated with DMEM without FBS for 48 h at 37 °C. The migration in HUVECs was observed using the microscopy (Nikon, Japan).

### Tube formation

The Matrigel was mixed with ECMatrix diluent buffer onto the 96 well plates. After the mixed solution fixation, HUVECs were seeded onto the plate with DMEM under 37℃ at 5% CO_2_ for 12 h. Tube formation was overserved by 100 × microscope. The number in three visual fields randomly was calculated as average.

### Hematoxylin–eosin staining

The wound tissue samples from DFU mice were immersed in 10% neutral formalin over day. These samples were stained with hematoxylin and eosin after the management of xylene and ethanol.

### CD31 Immunohistochemistry

Immunohistochemistry of tissue sections with rabbit anti-mouse CD31 antibody (Santa Cruz Biotechnology, USA) was performed at 7 days to detect angiogenesis in the wound after intervention. Sections were deparaffinized, rehydrated, heated in a microwave oven twice for antigen recovery, treated with 3% H_2_O_2_ and then incubated with 5% goat serum albumin. Then, the sections were incubated overnight at 4 °C with primary rabbit anti-mouse CD31 antibody (1:75, Santa Cruz Biotechnology), followed by a 1 h incubation with HRP-conjugated goat anti-rabbit secondary antibody (1:200, Abcam). After being counterstained with hematoxylin, the slides were assessed with a fluorescence microscope (40FL Axioskop, Zeiss).

### Flow cytometry

HUVECs were seeded onto 6-well plate at the density of 100,000 cell per well. After 48 h, cells were harvested using trypsin (Gibco, USA) to be incubated with Annexin V-FITC and propidium iodide (PI) which were purchased from Procell company (China) for 25 min at dark. Apoptosis rate of HUVECs was determined by flow cytometry (BD biosciences, USA).

### Statistics and analysis

SPSS 22.0 (IBM, USA) was used for data difference analysis and Graphpad 8.0 (USA) was used for data picture drawing. Comparison between two groups was processed with independent sample t test. One-way ANOVA was used to analyze the differences among groups. Afterward, post-pairwise comparison was conducted by Dunnett’s multiple comparison tests. Comparison among multiple groups at different time points was analyzed by repeated measures ANOVA. All comparisons were two-sided tests. 95% was taken as the confidence interval and the difference was statistically significant when P value was less than 0.05. In this article, the experiment was repeated three times. Each animal group consisted of 5 of animals.

## Results

### The characterization of ADSCs and exosomes

**Fig. 1:**
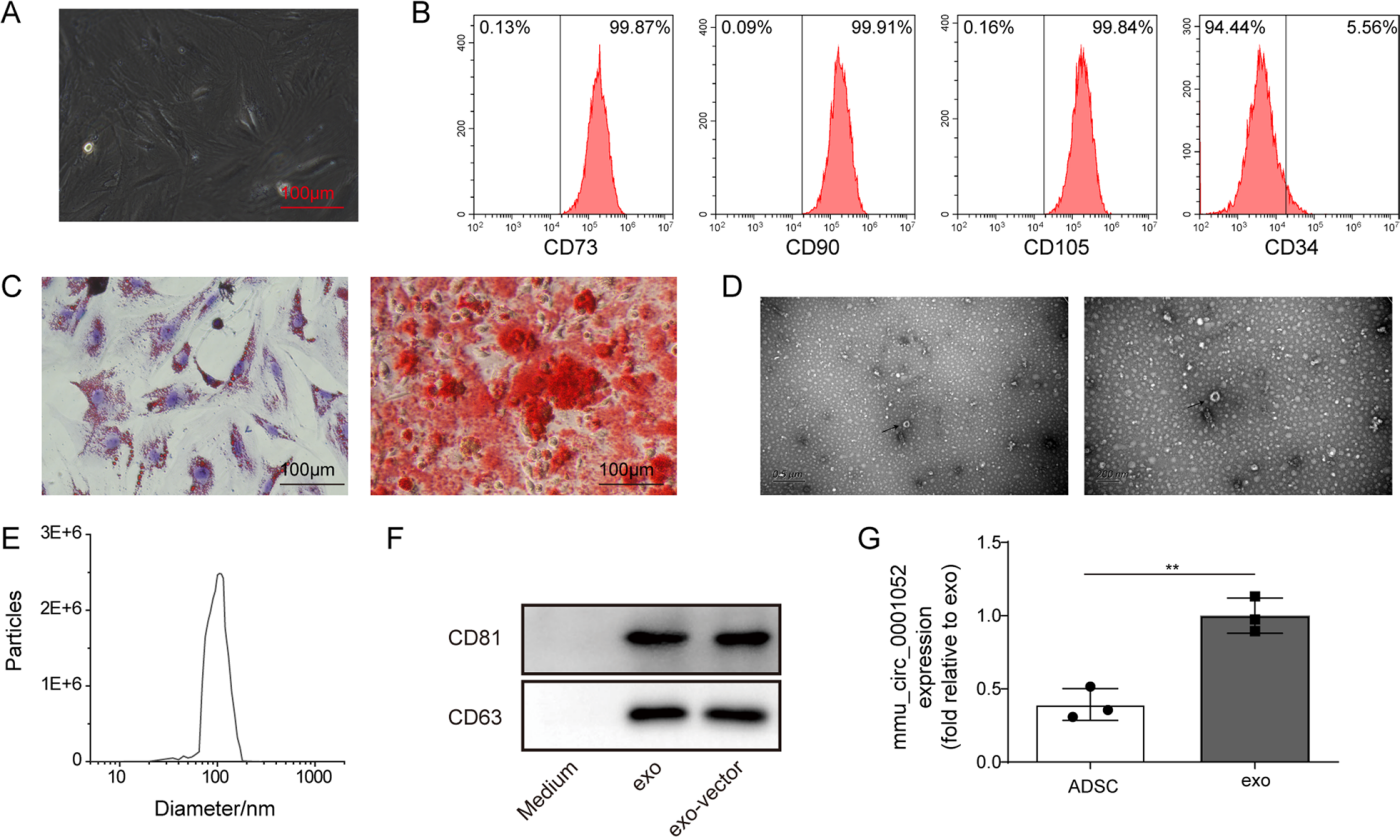
The characterization of ADSCs and exosomes. **A** morphology of ADSCs. **B** detection of surface markers of ADSCs by flow cytometry. **C** the osteogenic and adipogenic differentiation via oil red o and alizarin red. **D** appearance of exosomes via transmission electron microscope. **E** diameter of exosomes via dynamic light scattering. **F** biomarkers (CD63 and CD81) via western blot. **G** the expression of mmu_circ_0001052 in ADSCs and exosomes via real-time PCR. All data were obtained from at least three replicate experiments. *P < 0.05, **P < 0.01. Medium mean the medium after extracting exosomes. N = 3. Comparison between two groups was processed with independent sample t test. One-way ANOVA was used to analyze the differences among three groups. Afterward, post-pairwise comparison was conducted by Dunnett’s multiple comparison test

As previous studies have been suggesting that ADSCs can ameliorate the wound injury in DFU, we focused on the specific molecular mechanism of ADSCs-derived exosomes in DFU. Firstly, ADSCs were isolated from adipose tissue and cultured in DMED for three weeks. ADSCs were spindle-shaped (Fig. 1A). The surface antigens of ADSCs were analyzed by flow cytometry. As illustrated in Fig. 1B, CD73(99.87%), CD90(99.91%) and CD105(99.84%) were positive, whereas CD34(5.56%) was negative. The result from flow cytometry showed these cells had specific biomarkers of ADSCs. Then, we observed the osteogenic and adipogenic differentiation of ADSCs via oil red O and alizarin red. Figure 1C reveals that ADSCs could differentiate into adipocytes or osteoblasts. The exosomes (purity: 3 × 10^9^ particles/mL) were isolated from ADSC and characterized by transmission electron microscope and western blot. The ADSCs-derived exosomes were, with diameter ranging from 30 to 200 nm, round shape, saucer shape or hemispherical double-layer pattern structure with one-sided depression (Fig. 1D and E). The surface biomarkers of exosomes were determined by western blot, of which result displayed that CD63 and CD81 were detected in these exosomes (Fig. 1F). Finally, we found that mmu_circ_0001052 was expressed in both ADSCs and exosomes, and was abundantly expressed in exosomes (Fig. 1G).

### Overexpression mmu_circ_0001052 in ADSCs-derived exosomes promoted proliferation, migration and angiogenesis of high glucose-induced HUVECs

**Fig. 2:**
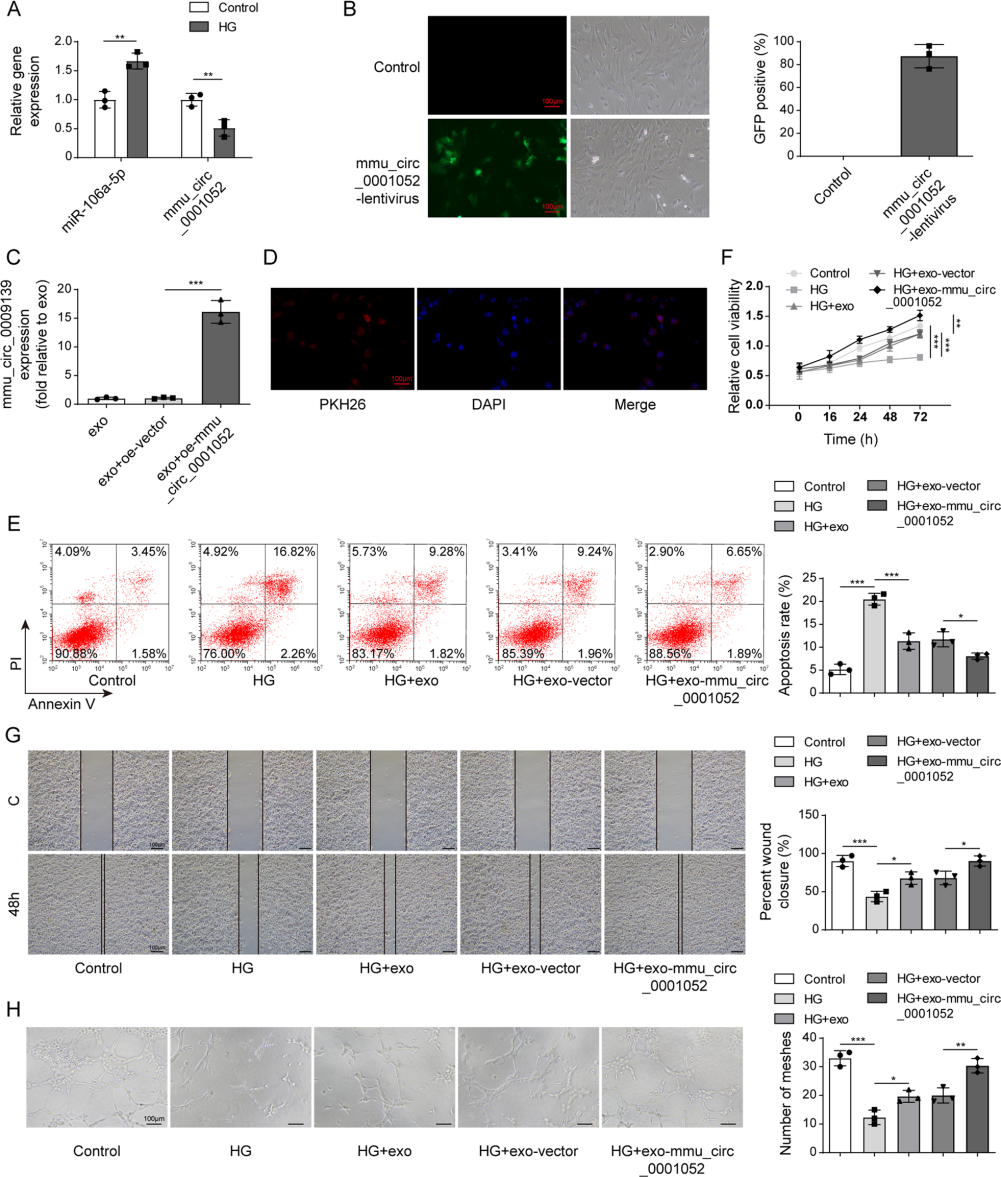
Overexpression mmu_circ_0001052 in ADSCs-derived exosomes promoted proliferation, migration and angiogenesis of high glucose-induced HUVECs. **A** the expression of miR-106a-5p and mmu_circ_0001052 in high glucose-induced HUVECs. **B** the transfection efficiency via fluorescence microscope. **C** the expression of mmu_circ_0001052 in ADSCs transfected with different vectors (exosomes in exo group was extracted from normal ADSCs, these in exo+oe-vector group from ADSCs transfected with empty vector, and these in exo+oe-mmu_circ_0001052 group from ADSCs transfected with oe-mmu_circ_0001052). **D** the fusion of exosomes into cells via immunofluorescence. **E** apoptosis by flow cytometry. **F** proliferation by MTT. **G** migration by transwell. **H** angiogenesis via tube formation assay. All data were obtained from at least three replicate experiments. *P < 0.05, **P < 0.01. N = 3. One-way ANOVA was used to analyze the differences among groups. Afterward, post-pairwise comparison was conducted by Dunnett’s multiple comparison test. Comparison among multiple groups at different time points was analyzed by repeated measures ANOVA

We overexpressed the expression of mmu_circ_0001052 in ADSC to increase its enrichment in exosomes that were performed to treating high glucose-induced HUVEC. Subsequently, cell processes were observed including proliferation, migration and angiogenesis. As shown in Fig. 2A, miR-106a-5p was increased whereas mmu_circ_0001052 decreased in high glucose-induced HUVECs. Figure 2B suggests it was approximately that lentivirus transfection efficiency of mmu_circ_0001052 overexpression vector was 80%. According to Fig. 2C, mmu_circ_0001052 was upregulated successfully in ADSCs. Figure 2D illustrates that ADSCs-derived exosomes could be absorbed by high glucose-induced HUEVCs and distributed around the nucleus, which implied ADSCs-derived exosomes could get into HUVECs and played the potential role.

In Fig. 2E, compared with HUVECs without high glucose (control group), high glucose-induced HUVECs (HG group) expressed the increase in apoptosis, whose apoptosis rate was about 19%; while the apoptosis rates of HG + exo group and HG + exo-vector group showed difference in statistics compared with HG group, that of HG + exo-mmu_circ_0001052 group markedly dropped to about 8.5%. Figure 2F shows that high glucose-induced the decreased proliferation of HUVECs; The proliferation of HG + exo group and HG + exo-vector group increased, but the difference was not statistically significant; HG + exo-mmu_circ_0001052 got the significant elevation of proliferation in HUVECs. The results of migration and angiogenesis showed the same trend. In Fig. 2G and 2H, high glucose led to decreased migration and weakened angiogenesis of HUVECs; exosome and exosomes plus vector could increase the migration and angiogenesis; HG + exo-mmu_circ_0001052 group displayed the most capability of migration and angiogenesis.

The above results suggested that high glucose resulted in the decrease in migration, proliferation and angiogenesis and the increase in apoptosis, but ADSCs-derived exosomes with upregulated mmu_circ_0001052 could improve these events.

### Overexpression of miR-106a-5p targeting FGF4 inhibited the proliferation, migration and angiogenesis of HUVECs induced by high glucose

**Fig. 3:**
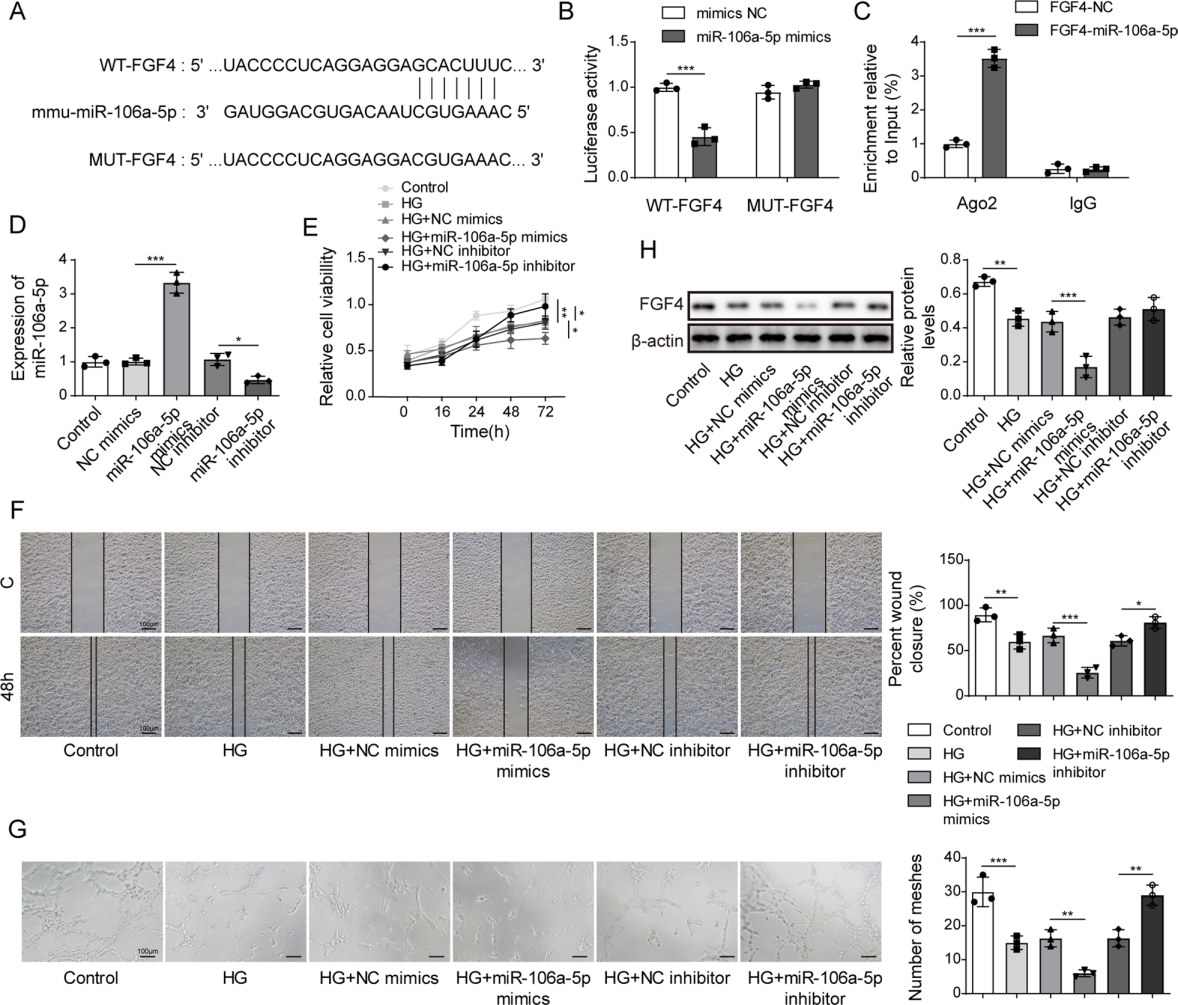
Overexpression miR-106a-5p targeting FGF4 inhibited proliferation, migration and angiogenesis of high glucose-induced HUVECs. **A** the target of miR-106a-5p and FGF4 via targetscan7.2. **B** Dual-luciferase reporter gene assay. **C** RNA binding protein immunoprecipitation. **D** the expression of miR-106a-5p via real-time PCR. **E** proliferation by MTT. **F** migration by transwell. **G** angiogenesis via tube formation assay. **H** the level of FGF4 via western blot. All data were obtained from at least three replicate experiments. *P < 0.05, **P < 0.01. *N *= 3. One-way ANOVA was used to analyze the differences among groups. Afterward, post-pairwise comparison was conducted by Dunnett’s multiple comparison test. Comparison among multiple groups at different time points was analyzed by repeated measures ANOVA

According to Fig. 2, miR-106a-5p was up-regulated in high glucose-induced HUVECs, which implied up-regulated miR-106a-5p might be involved in proliferation, migration and angiogenesis of HUVECs. Targetscan7.2 predicted that the 3’UTR of FGF4 existed the binding site to miR-106a-5p (Fig. 3A). According to this predicted site, we designed dual-luciferase reporter gene experiment and RIP assay to verify the target relationship between miR-106a-5p and FGF4. In Fig. 3B and C, the relative luciferase activity decreased when cells were co-transfected by miR-106a-5p mimics and WT FGF4; miR-106a-5p was highly enriched in FGF4. These results determined that miR-106a-5p could bind to FGF4 via the site. The overexpression of miR-106a-5p was significantly higher than the control group and NC mimics group, indicating that miR-106a-5p was successfully transfected (Fig. 3D).

As shown in Fig. 3E, high glucose inhibited the proliferation of normal HUVECs; subsequently up-regulated miR-106a-5p repressed the proliferation of high glucose-induced HUVECs. On the contrary, miR-106a-5p downregulation enhanced that of high glucose-induced HUVECs (Fig. 3E). Moreover, high glucose resulted in the decrease in migration and angiogenesis in normal HUVECs, and overexpression of miR-106a-5p could inhibit migration and angiogenesis of the high glucose-induced HUVEC but miR-106a-5p caused the opposite results (Fig. 3F and G). FGF4 has been determined to be the target of miR-106a-5p. Therefore, the expression of FGF4 was decreased with miR-106a-5p increase although that of FGF4 was elevated due to miR-106a-5p downregulation (Fig. 3H).

The above results indicate that overexpression of miR-106a-5p inhibited the proliferation, migration and angiogenesis of HUVEC induced by high glucose via targeting FGF4.

### mmu_circ_0001052 in exosomes promoted proliferation, migration and angiogenesis of high glucose-induced HUVECs via miR-106a-5p and FGF4/p38MAPK pathway

**Fig. 4:**
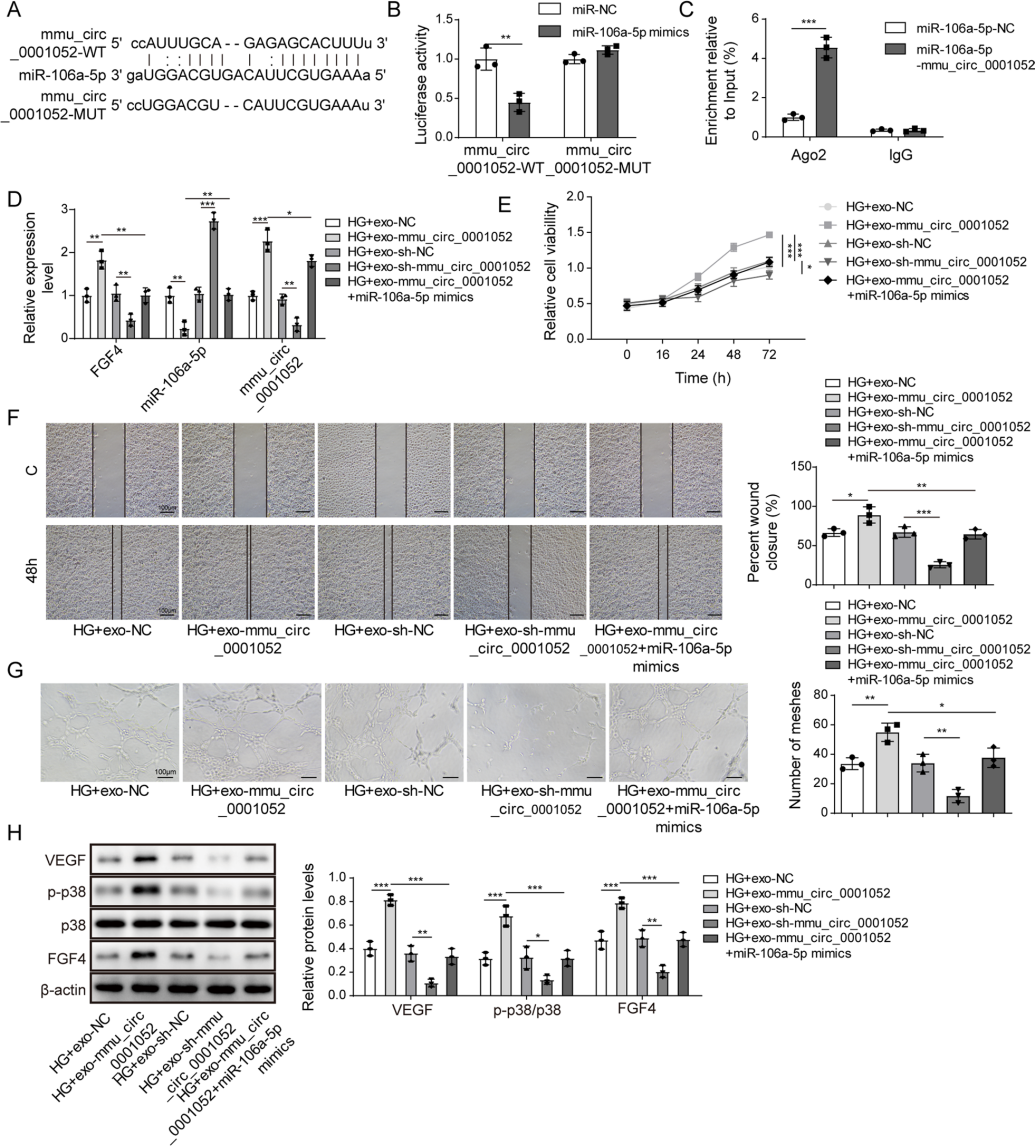
Mmu_circ_0001052 in exosomes promoted proliferation, migration and angiogenesis of high glucose-induced HUVECs via miR-106a-5p and FGF4/p38MAPK pathway. **A** the target of miR-106a-5p and mmu_circ_0001052 via starbase3.0. **B** Dual-luciferase reporter gene assay. **C** RNA binding protein immunoprecipitation. **D** The expression levels of miR-106a-5p, mmu_circ_0001052 and FGF4 via real-time PCR. **E** proliferation by MTT. **F** migration by transwell. G, angiogenesis via tube formation assay. **H** the level of FGF4/p38MAPK pathway via western blot. All data were obtained from at least three replicate experiments. *P < 0.05, **P < 0.01. *N* = 3. One-way ANOVA was used to analyze the differences among groups. Afterward, post-pairwise comparison was conducted by Dunnett’s multiple comparison test. Comparison among multiple groups at different time points was analyzed by repeated measures ANOVA

CircRNAs regulated via targeting miRNAs. Given that the trends of miR-106a-5p and mmu_circ_0001052 in high glucose-induced HUVECs, we inferred miR-106a-5p might be the target of mmu_circ_0001052. The binding relationship between miR-106a-5p and mmu_circ_0001052 has been predicted via starbase3.0. The 3’UTR of mmu_circ_0001052 existed one site binding to miR-106a-5p (Fig. 4A). Figure 4B suggests that the relative luciferase activity decreased when cells were co-transfected by miR-106a-5p mimics and WT mmu_circ_0001052. The enrichment of miR-106a-5p on mmu_circ_0001052 showed the phenomenon that mmu_circ_0001052 could directly bind to miR-106a-5p (Fig. 4C). Therefore, we proved miR-106a-5p was the target of mmu_circ_0001052. The increase in mmu_circ_0001052 resulted the decrease in miR-106a-5p and the increase in FGF4 (Fig. 4D). Moreover, upregulating miR-106a-5p could reverse the differential expression induced by mmu_circ_0001052 overexpression.

Increased mmu_circ_0001052 could improve proliferation of high glucose-induced HUVECs, but decreased mmu_circ_0001052 played the opposite role; the simultaneous upregulation of miR-106a-5p and mmu_circ_0001052 led to proliferation reduced compared with HG-exo-mmu_circ_0001052 group (Fig. 4E). Figure 4F and G shows that mmu_circ_0001052 ameliorated the migration and angiogenesis of HUVECs induced by high glucose, which could be reversed by increased miR-106a-5p. Previous studies indicated that FGF4 was involved in the activation of p38MAPK pathway. As illustrated in Fig. 4H, upregulating mmu_circ_0001052 resulted in the increase in VEGF and FGF4 and induced the phosphorylation of p38, whereas downregulating mmu_circ_0001052 worked on the opposite effects; upregulating miR-106a-5p reversed the differential trend induced by mmu_circ_0001052 overexpression.

The above results indicated mmu_circ_0001052 promoted proliferation, migration and angiogenesis of high glucose-induced HUVECs via miR-106a-5p and FGF4/p38MAPK pathway.

### Mmu_circ_0001052 from ADSCs-derived exosomes promoted wound healing in DFU via miR-106a-5p and FGF4/p38MAPK pathway

**Fig. 5:**
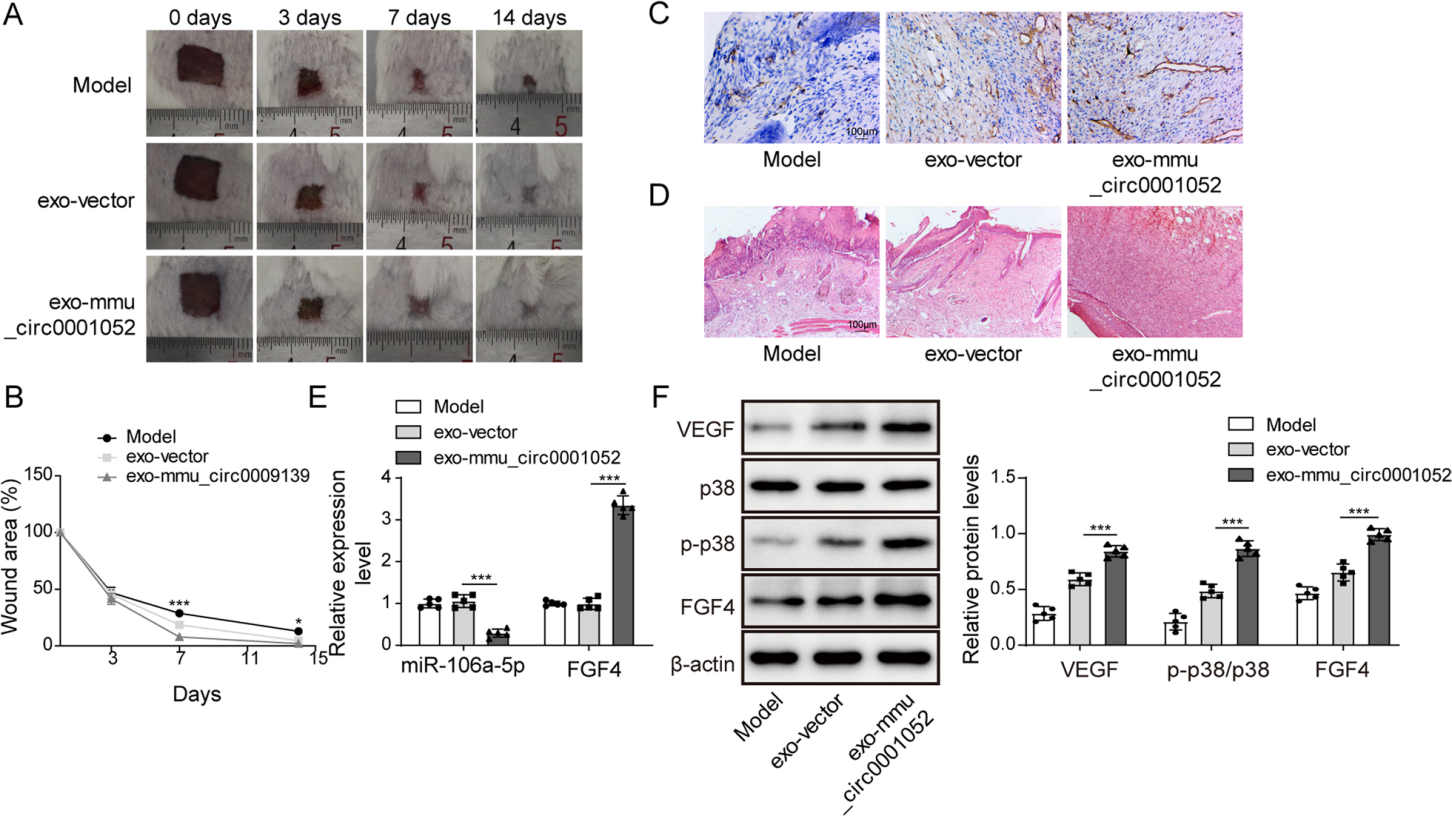
Mmu_circ_0001052 from ADSCs-derived exosomes promoted wound healing in DFU via miR-106a-5p and FGF4/p38MAPK pathway. **A** wound healing in DFU with exosomes treatment. **B** the broken line diagram of wound healing in DFU mice. **C** the vessel formation under Immunofluorescence. **D** HE staining. **E** the expression of miR-106a-5p and FGF4 in DFU mice. F, the level of FGF4/p38 pathway via western blot. All data were obtained from at least three replicate experiments. *P < 0.05, **P < 0.01. *N* = 5. One-way ANOVA was used to analyze the differences among groups. Afterward, post-pairwise comparison was conducted by Dunnett’s multiple comparison test. Comparison among multiple groups at different time points was analyzed by repeated measures ANOVA

We designed a mice model of DFU to determine the role of mmu_circ_0001052 from ADSCs-derived exosomes in wound healing. Compared with model group, exo+vector group and exo+mmu_circ_0001052 group showed the obvious wound healing; exo+mmu_circ_0001052 group displayed the faster speed of healing and the smaller area of wound compared with exo+vector group (Fig. 5A and B). As shown in Fig. 5C, exo+vector and exo+mmu_circ_0001052 promote the formation of capillary; the effect of exo+mmu_circ_0001052 was better than exo+vector. The exosomes with upregulated mmu_circ_0001052 promoted wound healing in DFU via diminishing inflammatory cells and promoting granulation tissue (Fig. 5D). The expression of miR-106a-5p and FGF4 showed non-statistical difference after the treatment of exosome containing vector in mice with DFU, but decreased miR-106a-5p and increased FGF4 were observed in DFU mice treated with exosomes containing upregulated mmu_circ_0001052 (Fig. 5E). Finally, the level of FGF4/p38MAPK pathway was detected by western blot. Figure 5F reveals that exosomes containing upregulated mmu_circ_0001052 led to the higher level of FGF4, VEGF and phosphorylation of p38, suggesting that exosomes containing upregulated mmu_circ_0001052 could promote wound healing in DFU via FGF4/p38MAPK pathway. To conclude, a mechanism was determined that mmu_circ_0001052 from ADSCs-derived exosomes promoted wound healing in DFU via miR-106a-5p and FGF4/p38MAPK pathway.

## Discussion

DFU, a diabetic complication, is caused by poor glycemic control, peripheral neuropathy, peripheral vascular disease and immunosuppression. Metabolic disorder induced by hyperglycemic status leads to vascular dysfunction and nerve injury in vivo, which subsequently causes the foot ulcers and lower limb infection [23]. ADSCs, a MSCs derived from adipose, is confirmed to promote wound healing through vessels formation. Li [21] concluded the mechanism that exosomes derived from ADSCs enhanced, via Nrf2, the proliferation and angiogenesis of endothelial progenitor cells to finally ameliorate wound healing in DFU, which implied that ADSCs-derived exosomes had considerable utilization value in DFU management.

CircRNAs enriched in exosomes regulates the process of diseases through endocytosis and exocytosis. A previous study by Xu et al. [24] found exosomal circAKAP7 derived from ADSCs the cellular damage and oxidative stress induced by cerebral ischemia. Zhu et al. [24] published the viewpoint that ADSCs-derived exosomes repressed liver fibrosis by autophagy induced by mmu_circ_0000623. We realized that exosomal mmu_circ_0001052 derived from ADSCs could be absorbed into high glucose-induced HUVESs, which contributed to the proliferation, migration and angiogenesis of HUVECs. In our results, Mmu_circ_0001052 was admitted to be decreased in high glucose-induced HUVECs. Mmu_circ_0001052 is a homolog of circRNA HIPK3 in mice which is considered as the role functioning cardiovascular disease and diabetes. CircRNA HIPK3 elevated the proliferation of cardiomyocyte via acetylating Notch1, which subsequently improved myocardial damage and fibrosis [25]. CircRNA HIPK3 functions as the inducer of proliferation and angiogenesis of HUVECs induced by high glucose [12, 26]. To conclude, the result that exosomal mmu_circ_0001052 derived from ADSCs indicated that mmu_circ_0001052 overexpressed in ADSCs was absorbed into HUVECs induced by high glucose to promote the vessels formation of cells.

We found that high glucose increased miR-106a-5p in HUVECs. MiR-106a-5p, a member of miRNA family, plays the molecular regulatory role in multiple sclerosis, colorectal cancer and other chronic diseases [27–29]. Commonly, mmu_circ_0001052 could be involved in cell process by targeting miRNAs. The trend of miR-106a-5p, different from mmu_circ_0001052 in high glucose-induced HUVECs, suggested the hypothesis that there was a targeting regulatory relation between miR-106a-5p and mmu_circ_0001052. MiR106a-5p was confirmed to be the target of mmu_circ_0001052 by bioinformatics tool and experiments. Afterward, we discovered that FGF4 was the targeting gene of miR-106a-5p. When miR-106a-5p was upregulated in high glucose-induced HUVECs, there was the downregulation of FGF4 accompanied with the decrease in proliferation, migration and angiogenesis, which implied that miR-106a-5p repressed the vessel formation of high glucose-induced HUVECs via targeting FGF4.

FGF4 is related through p38MAPK pathway regulating the biological function of endothelial cells in cell differentiation [20], which shows that FGF4/p38MAPK pathway acted on the vessel formation of HUVECs induced by high glucose. A previous study [30] showed that FGF4 could improve wound healing in DFU with VEGF-A. Ramirez concluded that FGF was involved in wound healing in DFU. Subsequently, we carried out the research into the relationship among mmu_circ_0001052, miR-106a-5p and FGF4/p38MAPK pathway. Increased miR-106a-5p reversed the trend caused by exosomal mmu_circ_0001052 that FGF4 was increased and p38MAPK pathway activated in high glucose-induced HUVECs. Meanwhile, the facilitations of exosomal mmu_circ_0001052 on vessel formation was weaken with FGF4 decreased. These results suggested that exosomal mmu_circ_0001052 derived from ADSCs promoted the angiogenesis of high glucose-induced HUVECs through miR-106a-5p and FGF4/p38MAPK pathway. A model with DFU was established to determine the role of exosomal mmu_circ_0001052 from ADSCs on DFU treatment. We found exosomal mmu_circ_0001052 from ADSCs had better effect in promoting wounding healing and improving wound area. In molecular level, exosomal mmu_circ_0001052 obviously repressed miR-106a-5p and activated FGF4/p38MAPK. The results above indicated that exosomal mmu_circ_0001052 from ADSCs improved wound healing in DFU via targeting miR-106a-5p/FGF4/p38MAPK axis.

DFU is a complex and chronic disease, in which circRNA functions as the regulatory element via intricate network. Obviously, miR-106a-5p/FGF4/p38MAPK axis is not the only target of exosomal mmu_circ_0001052 in DFU. Therefore, next studies could probe other ways, such as miRNAs or signaling pathway, that exosomal mmu_cicr_0009139 was related to. Moreover, we failed to discuss the role of exosomal mmu_cicr_0009139 in the anthropogenic with DFU. It is feasible to determine the relationship between clinical characterizations of DFU and mmu_circ_0001052.

## Conclusions

We reported a novel mechanism of mmu_circ_0001052 in diabetic angiogenesis. Exosomes derived from mmu_circ_0001052-modified ADSCs promoted the angiogenesis of wound healing in DFU via miR-106a-5p and FGF4/p38MAPK pathway, which indicated that mmu_circ_0001052/miR-106a-5p/FGF4/p38MAPK pathway could be the potential target of DFU treatment.

## Supplementary Information

**Additional file 1.** The sequence of mmu_circ_0009139.

## Data Availability

All data generated or analyzed during this study are included in this article. The datasets used and/or analyzed during the current study are available from the corresponding author on reasonable request.

## Abbreviations

DFU: Diabetic foot ulcer
DM: Diabetes mellitus
ADSCs: Adipose-derived stem cells
FGF4: Fibroblast growth factor 4
MAPK: Mitogen-activated protein kinase
MSCs: Mesenchymal stem cells
PBS: Phosphate buffer saline
PCR: Polymerase chain reaction
HUVECs: Human umbilical vein endothelial cells
UTR: Untranslated region
DMEM: Dulbecco’s modified eagle’s medium
FBS: Fetal bovine serum
