# Supplementary material for: Exosomes from mmu_circ_0001052-modified adipose-derived stem cells promote angiogenesis of DFU via miR-106a-5p and FGF4/p38MAPK pathway
Source: Stem Cell Res Ther. 2022 Jul 23;13:336. doi: 10.1186/s13287-022-03015-7 (PMC9308214; doi:10.1186/s13287-022-03015-7)
Supplement: Supplementary file 1 — Additional file 1. The sequence of mmu_circ_0009139. [file 13287_2022_3015_MOESM1_ESM.docx]

>mmu_circ_0009139

GTATTCCTGCAGCACATGGTAAACCCACCAGTTATTCCATAAGGGTAGACAATACAGTTCCGCTTGTAACTCAGGCCCCAGCTGTGCAGCCTCTGCAGATCCGACCTGGAGTCCTTTCACAGCAGACATGGTCTGGTCGAACACAGCAGATGCTAATACCTGCCTGGCAGCAGGTAACACCCATGGCTCCTGCTGCCGCAACACTAACTTCTGAAGGCATGGCTGGTTCTCAGAGGCTTGGAGACTGGGGGAAAATGATTCCACACAGCAATCATTACAACTCGGTGATGCCACCGCCTCTTCTAACCAACCAGATCACATTATCAGCCCCTCAGCCTATCAGCGTGGGCATTGCACATGTTGTCTGGCCTCAGCCTGCCACTACCAAGAAAAATAAGTTGTGCCAGAACAGAGGTATTTTGGTAAGACTAATGGAATGGGAGCCAGGAAGAGAGGAAATAAATGCTTTCCGTTGGAGTAATTCATTGCAGAACACCAATATCCCACATTCAGCATTTATTTCTCCAAAGATAATCAGTGGGAAAGAGGTTGAGGAAGTAAGTTGTGTAGACACACAGGACAATCATACCTCAGAAGGAGAGGCCGGAACTTGCCGTGAAGCGTCTGTCAGACAGGATTCTTCAGTCTCAGACAAACAGCGGCAAACCATCATCATTGCCGACTCCCCGAGTCCTGCCGTGAGTGTGATCACCATTAGCAGTGACAGCGATGATGAAGAGACCTCACCCAGACCTTCACTCCGAGAGTGTAAAGGTAGTCTAGATTGTGAAGCTTGCCAAAGCACTTTGAATATTGATCGGATGTGTTCACTCAGCAGTCCTGATAGCACTCTGAGCACCAGCTCCTCAGGGCAGTCCAGCCCGTCCCCTTGCAAGAGACCGAACAG
